# Enhanced PEC performance of nanoporous Si photoelectrodes by covering HfO_2_ and TiO_2_ passivation layers

**DOI:** 10.1038/srep43901

**Published:** 2017-03-02

**Authors:** Zhuo Xing, Feng Ren, Hengyi Wu, Liang Wu, Xuening Wang, Jingli Wang, Da Wan, Guozhen Zhang, Changzhong Jiang

**Affiliations:** 1 Center for Ion Beam Application and Center for Electron Microscopy, School of Physics and Technology, Wuhan University, Wuhan 430072, People’s Republic of China; 2 Key Laboratory of Artificial Micro- and Nano-structures of Ministry of Education, School of Physics and Technology, Wuhan University, Wuhan 430072, People’s Republic of China

## Abstract

Nanostructured Si as the high efficiency photoelectrode material is hard to keep stable in aqueous for water splitting. Capping a passivation layer on the surface of Si is an effective way of protecting from oxidation. However, it is still not clear in the different mechanisms and effects between insulating oxide materials and oxide semiconductor materials as passivation layers. Here, we compare the passivation effects, the photoelectrochemical (PEC) properties, and the corresponding mechanisms between the HfO_2_/nanoporous-Si and the TiO_2_/nanoporous-Si by I–V curves, Motte-schottky (MS) curves, and electrochemical impedance spectroscopy (EIS). Although the saturated photocurrent densities of the TiO_2_/nanoporous Si are lower than that of the HfO_2_/nanoporous Si, the former is more stable than the later.

The need for clean, safe, and sustainable energy has always been one of most important challenges in today’s world, since worldwide energy demand will increase 37% in the next 20 years. With the adjustment of global energy framework, people pay more attention to new energy, and it has to meet more severe criteria: renewable, environment-friendly, high-energy density. Hydrogen meets all requirements above. The credible decarbonized fuel is produced from water, and then water is generated without toxic gas after the fuel combustion for releasing large amounts of energy in a sustainable way.

Harvesting hydrogen energy directly from inexhaustible sunlight with minimal environment impacts offers an ideal way to solve energy and climate crisis. Liking storage solar energy by plants through photosynthesis, semiconductors can store energy in chemical bands by photochemical catalysis. Photoanode materials should have the suitable valence band (VB) that are more positive than the potential of *E*^*0*^ (O_2_/H_2_O) for oxygen evolution. Furthermore, photoanode materials must be stable under oxidation and illumination in aqueous. Some metal oxides, including TiO_2_, WO_3,_ and ZnO, with the VB that consists of O 2p orbitals meet these requirements^1^,^2^,^3^. Many p-type semiconductors which have more negative conduction band (CB) than the potential of *E*^*0*^ (H_2_/H_2_O) are investigated as photocathode materials for H_2_ evolution, including GaP, Cu_2_O, p-Si^4^,^5^,^6^. The stability of photocathode materials in aqueous is very important, which can provide continuous photocurrent under illumination.

As one of the most promising photoelectrode materials, silicon (Si) has many advantages. Si has gained the extensive acceptance due to the excellent electrical properties, the superior thermostability of electrical properties, low cost, and so on. Owing to the large diffusion coefficient (*D*_*n*_ = 34.6 and *D*_*p*_ = 12.3 cm^2^/s for electrons and holes, respectively) and the long lifetime of carries (τ = 130 μs), the diffusion length of charge carriers in Si can reach about 1μm even the doping density as high as 10^19^~10^20^ cm^−3^. In recent research, the photocurrent density and efficiency of Si are as high as 30 mA/cm^2^ and 4.9%, respectively^7^.

However, the disadvantages of Si as the photoelectrode material are also not negligible. The bandgap of Si is 1.12 eV, which is not large enough for full water splitting^8^. Hence, Si is used as the photocathode since its suitable CB position for hydrogen generation^9^,^10^. Moreover, a more pivotal problem is that Si is easy to be oxidized in any aqueous photoelectrochemical (PEC) system. It can actively react with water to produce H_2_ (gas), SiH_4_, silicate and SiO_2_, since the standard potential of Si is −0.857 V vs. standard hydrogen electrode (SHE). The thickness of oxide layer on Si depends on the original surface condition, the reaction time, the pH of solution, the dissolved oxygen, etc^11^. In the real application, the cathode bias voltage that generated by illumination is not effective enough to protect the surface of Si from oxidation. Any uncontrollable oxide layer caused by this unavoidable effect will prevent H^+^ reduction. Therefore, the performance of Si photoelectrodes will decay quickly.

Recently, nanostructured Si shows enhanced PEC performances, while it also brings more severe challenges of stability in aqueous. One of the main gains for nanostructure is the enhanced surface area, which improves the absorption of light, the collection of charge carriers, the increase of exchange current densities, and the desorption of gas. However, the surface of that nanostructure photoelectrode is more likely to be oxidized. A very thin oxide layer that was presented on the surface of the delicate nanostructure will have an enormous impact on the PEC properties.

Facing above challenges, many strategies have been applied to avoid the disadvantage of Si PEC system. Capping a passivation layer on the surface of Si is one of the most effective methods. Firstly, passivation layers can spatially separate the Si photoelectrodes from the severe working conditions in aqueous. Secondly, the composite structure can adjust the energy band structure vs. reversible hydrogen electrode (RHE) and the absorption of light. Meanwhile, the appropriate passivation layer itself possesses the high thermodynamically stability in aqueous and good pH-independent stability to be confronted with the rigorous and changeable work conditions.

Different passivation materials have been studied to achieve the highest performance of Si PEC photoelectrodes. Two kinds of passivation materials were especially paid attention. The first one is the insulating material with high dielectric constant, low light absorption. The representative materials are HfO_2_, ZrO_2_, Al_2_O_3_, etc. Due to its high thermodynamically stability and high refractive index, HfO_2_ becomes the promising candidate as passivation coating for Si. P.K. Singh *et al*. obtained a passivation layer with the surface recombination velocity less than 100 cm/s by atomic layer deposition (ALD) of HfO_2_^12^.

Different from the insulator, the oxide semiconductors have high anode corrosion potential, wide absorption of solar spectra light, and they are another kind of passivation materials. Typical oxide semiconductors passivation materials, e.g. TiO_2_, WO_3_, and SrTiO_3_ have ability to adjust energy band structure of composite PEC electrodes^13^. I. Chorkendorff *et al*. used high power impulse magnetron sputtering to deposit TiO_2_ on the surface of p-Si photocathode for H_2_ generation. Only a small degradation of photocurrent density was observed after a long-term stability test for 24 h^14^. The standard potential for anodic dissolution, 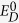, vs. the oxidation-reduction potential of electron acceptor in aqueous, 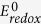, determines the stability of semiconductor in aqueous under long-term sunlight irradiation^15^.

However, all the above experimental results in different reported references are uncomparable in different material systems, and it is difficult to figure out which is the better kind of passivation layer. Moreover, there are few reports on the PEC stability studies of HfO_2_ passivation layers that covered on Si. Few works were also done to compare the different roles of HfO_2_ and TiO_2_ in these PEC systems. Figuring out the different passivation mechanisms of these two kinds of layers will help to understand the activities of electrons and holes during water splitting progresses, and then, assist the design of amenable PEC electrodes for practical application in the future. In this paper, the PEC properties of the HfO_2_/nanoporous-Si, represented the insulating layer, and the TiO_2_/nanoporous-Si, represented the oxide semiconductor layer, were analyzed, and compared by electrochemistry measurements, including I–V curves, Motte-schottky (MS) curves, and electrochemical impedance spectroscopy (EIS). The stabilities of the HfO_2_/nanoporous-Si and the TiO_2_/nanoporous-Si were also compared by long-term I-t curves.

## Results

### Morphology

**Figure 1:**
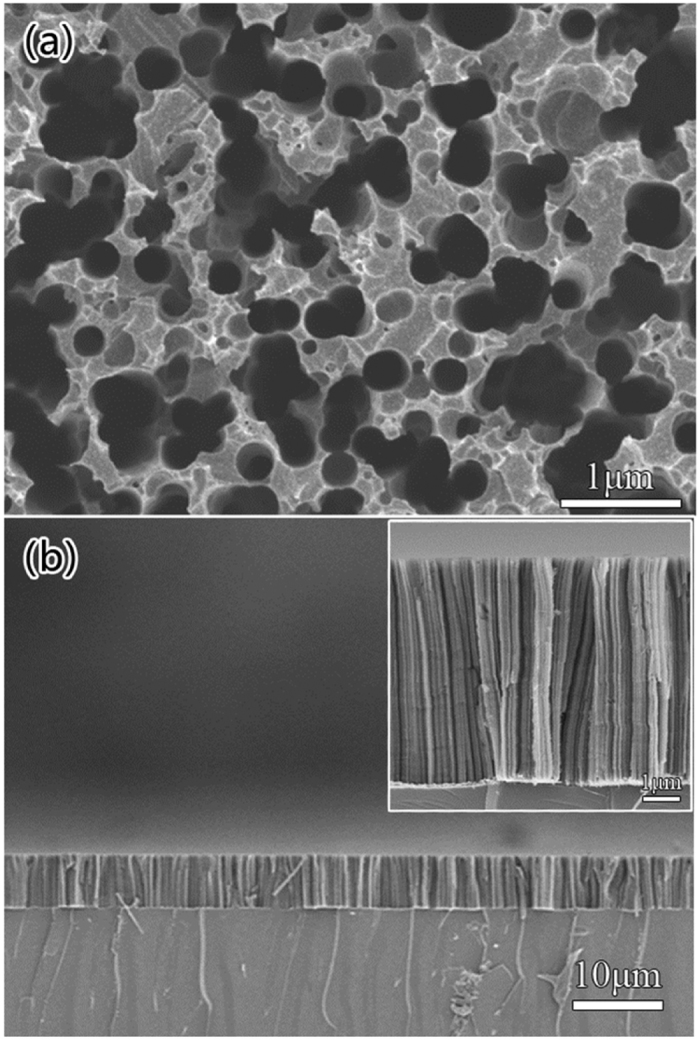
The planar (**a**) and the cross sectional (**b**) SEM images of the nanoporous Si.

MaCE is a low-cost and simple method to fabricate nanostructured Si, which can control the morphology of nanoporous by changing the etching time, the thickness of Au film, the recipe of etching solution and so on^16^. Figure 1 is the planar and cross-sectional SEM images of nanoporous Si (NP-Si). As shown in Fig. 1(a), the porous structure with diameter of 330 nm is obtained. Some holes are merged into one, which increases the diameter to 700~900 nm. Figure 1(b) demonstrates that a large scale of straight and ordered porous array is obtained. The inset image is the magnified cross-sectional SEM images of the NP-Si. No obvious change on the morphology of nanoporous Si after ALD deposition was observed. It shows that the length of the porous array is 6 μm. The nanoporous structure will facilitate the transfer and reaction of electrons on the inner wall of the nanoholes. In theoretically, the charge carrier transfer efficiency can reach the maximum only when the diffusion distance of charge carriers and the thickness of depletion layer are larger than the thickness of hole wall, and the hole depth is larger than the absorption depth of charge carriers.

**Figure 2:**
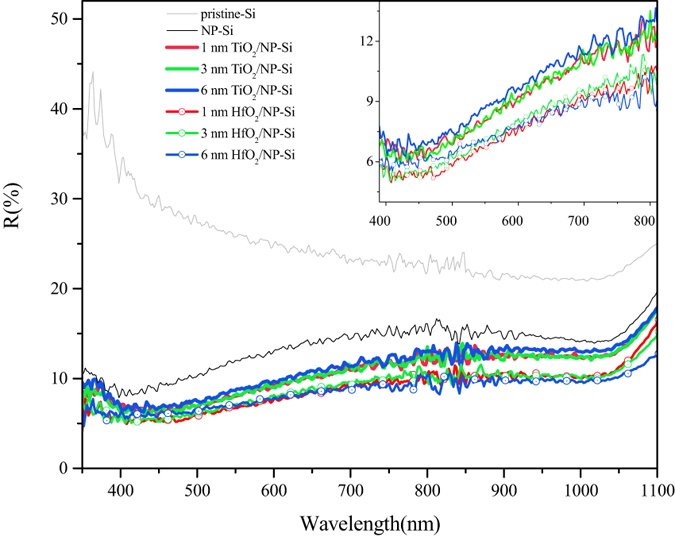
Reflection spectra of the pristine-Si (gray line), the NP-Si (black line), the HfO_2_(x)/Si (line) and the TiO_2_(x)/Si (line + symbol) from 350 to 1100 nm with step of 10 nm. The thicknesses of passivation layers are 1 (red), 3 (green), 6 (blue) nm, respectively.

For non-transparent simples, optical reflection can partially present the light absorption of samples. Figure 2 shows the reflection spectra of the pristine-Si, the NP-Si, the HfO_2_/NP-Si and the TiO_2_/NP-Si with different thicknesses of passivation layers. Owing to the nanoporous structure, the NP-Si has a less reflection in the ultraviolet and visible light area than that of the pristine-Si. The optical reflection spectra of both the HfO_2_/NP-Si and the TiO_2_/NP-Si change a little with increase in the thickness of the passivation layers. However, comparing to the TiO_2_/NP-Si, a low reflection of the HfO_2_/NP-Si is presented from 500 to 1100 nm, which can be corroborated by the smaller reflection index of HfO_2_ (n[HfO_2_] = 2.09)^17^ than that of TiO_2_ (n[TiO_2_] = 2.37~2.48)^18^. Reduction of optical reflection indicates the increase of optical absorption by the NP-Si, which may produce more charge carriers.

### PEC properties

**Figure 3:**
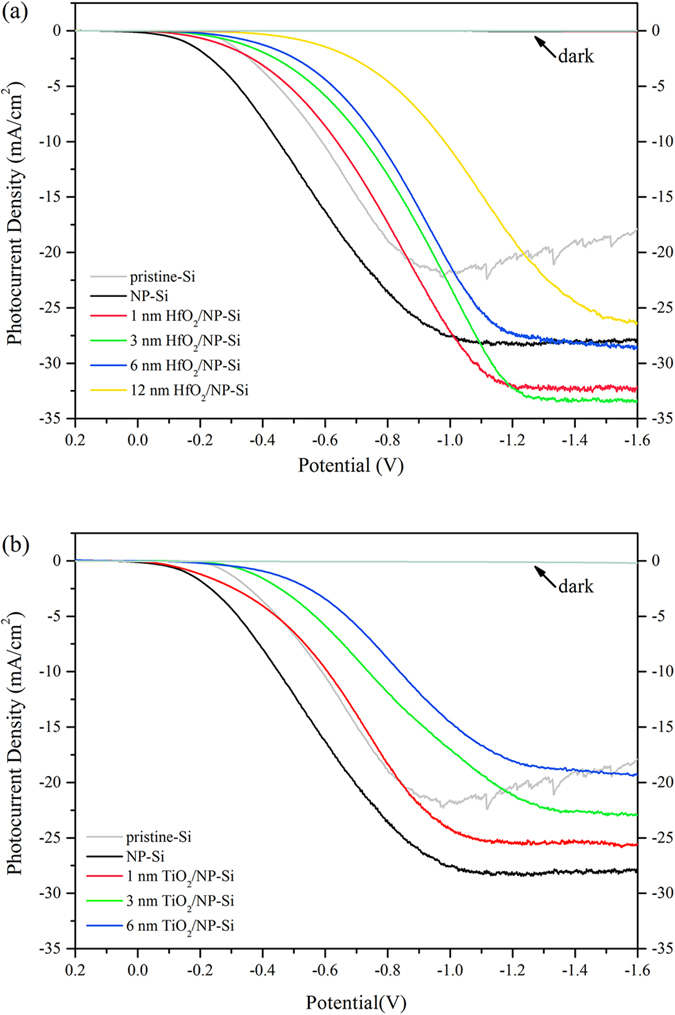
I–V curves of the pristine-Si (gray), the NP-Si (black), the HfO_2_(x)/NP-Si (**a**), and the TiO_2_(y)/NP-Si (**b**). The thickness of passivation layers x = 1 (red), 3 (green), 6 (blue), 12 (yellow) nm; y = 1 (red), 3 (green), 6 (blue) nm.

Figure 3 is the I–V curves between the pristine-Si, the NP-Si, the HfO_2_/NP-Si and the TiO_2_/NP-Si with the different thicknesses of passivation layers. Figure 3(a) illustrates the variation of photocurrent densities of the HfO_2_/NP-Si with increase in the thickness of the HfO_2_. The photocurrent densities of all the HfO_2_/NP-Si and the NP-Si are higher than that of the pristine-Si. The saturated photocurrent density of the NP-Si is nearly 10 mA/cm^2^ higher than that of the pristine-Si, which was benefited from the nanoporous structure. When the thickness of HfO_2_ increase from 1 to 3 and 6 nm, the saturated photocurrent densities increase firstly and decrease later. For the insulating and thin HfO_2_ film, there are two main mechanisms for forming currents. Within the effective thickness of HfO_2_ layer, tunneling effect is the dominate mechanism for the transfer of electrons, and the effective thickness is believed to be less than 3 nm. When the layer thickness is larger than the effective thickness, electrons jumping between defects and penetrating through the layer is the main source of current. In addition, the current decreases with the increase of the thickness of the HfO_2_ layer. The highest saturated photocurrent density achieves 33.5 mA/cm^2^ with the thickness of 3 nm in the HfO_2_/NP-Si. Then further increase the layer thickness of HfO_2_ to 12 nm, the saturated photocurrent densities keep decreasing. In Fig. 3(b), saturated photocurrent densities decrease obviously with increase of the layer thickness of TiO_2_. The saturated photocurrent density of 1 nm TiO_2_/NP-Si is 25.7 mA/cm^2^, which is lower than that of the NP-Si. It is worth noting that, both for the HfO_2_/NP-Si and the TiO_2_/NP-Si, before reaching the saturated photocurrent density, the photocurrent densities decrease with the increase of the thickness of the passivation layer at the same bias voltage (such as −0.8 V). This phenomenon is because the speed of electrochemical reaction is slower than the speed of charge carriers’ transfer in electrode, which means that the electrochemical polarization dominates the electrode process.

**Figure 4:**
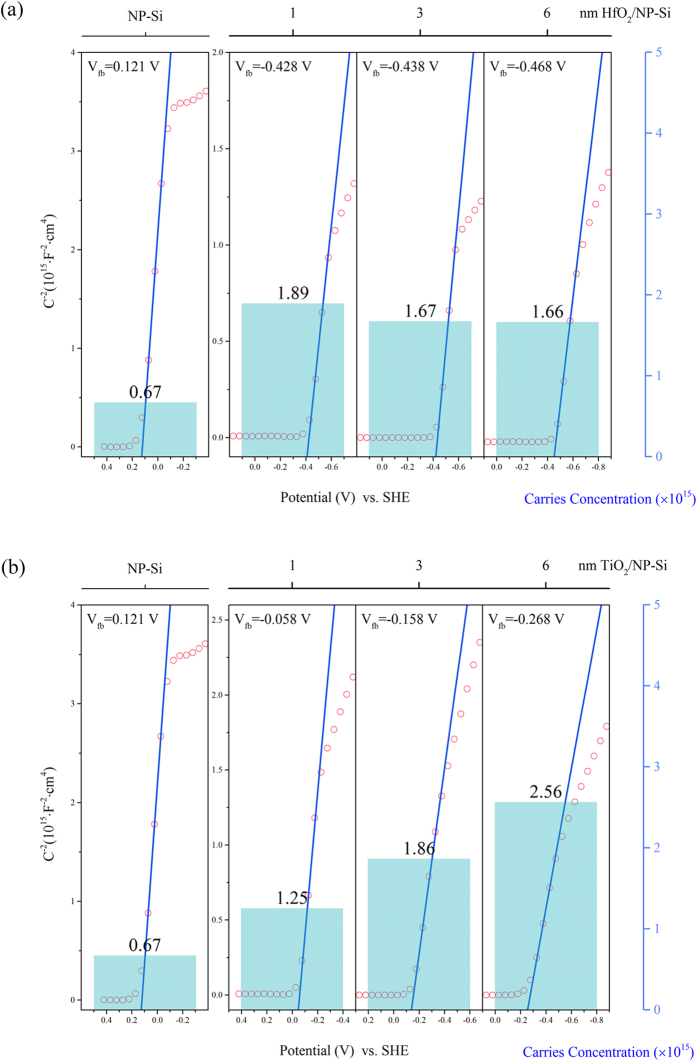
Mott-Schottky curves and corresponding carrier concentrations and flat band potentials vs. SHE of the NP-Si, the HfO_2_/NP-Si (**a**) and the TiO_2_/NP-Si (**b**) with the different thicknesses.

In order to figure out the influence of the passivation layers to the PEC properties, a series of measurements were carried out. Figure 4 is the comparison of Mott-Schottky (MS) curves between the pristine-Si, the HfO_2_/NP-Si and the TiO_2_/NP-Si with the different thicknesses of passivation layers, the corresponding carrier concentrations and flat band potentials are also presented. The calculation for the carrier concentrations as follows:


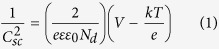


where *C*_*sc*_ is capacitance density, *e* is the charge of electron, *ε* is the permittivity of Silicon, *ε*_0_ is the permittivity of vacuum, *N*_*d*_ is carriers concentration, *V* is applied bias, *k* is the Boltzmann constant, *T* is Temperature. The x-intercept of the Mott-Schottky plot is reached at the bias that needs to be applied to cause the bands to become flat, and the x-intercept plus 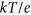 equals the flat band potential.

As shown in Fig. 4(a), compared with the NP-Si, the HfO_2_/NP-Si has higher carrier concentration, which means the HfO_2_ layer assists the gathering of charge carriers. The flat band potential of the HfO_2_/NP-Si is elevated to a more negative potential that close to the CB of Si than that of the NP-Si, which means the bending of CB at the interface of the HfO_2_/NP-Si is smaller than that of the electrolyte/NP-Si. With increase in the thickness of HfO_2_ layer, the carrier concentrations and flat band potentials of the HfO_2_/NP-Si have a small change. Because the HfO_2_ passivation layer is an insulator, the charge is fixed in the HfO_2_. The interface properties of the HfO_2_/NP-Si have larger influence on the carrier concentration and flat band potential than the thickness of the HfO_2_ layer. Different from the insulation layer of HfO_2_, TiO_2_ layer, as a semiconductor passivation layer, can greatly affect the space charge layer at the interface of TiO_2_/NP-Si. Then the carrier concentration and the flat band potential of the TiO_2_/NP-Si are dependent on the thickness of the TiO_2_ layer. As presented in Fig. 4(b), the carrier concentration in space charge layer increases with the thickness of TiO_2_ monotonically, the contribution of the carrier concentration is not only from the NP-Si, but also from the TiO_2_ layer. The flat band potential is elevated to −0.27 vs. SHE when the thickness of TiO_2_ is 6 nm, which is close to the CB of TiO_2_. Benefiting from the deposited passivation layer, the carrier concentration and flat band potential are increased in both the HfO_2_/NP-Si and the TiO_2_/NP-Si. However, the dependence of the flat band positions and the carrier concentrations on the thickness of passivation layers are different.

**Figure 5:**
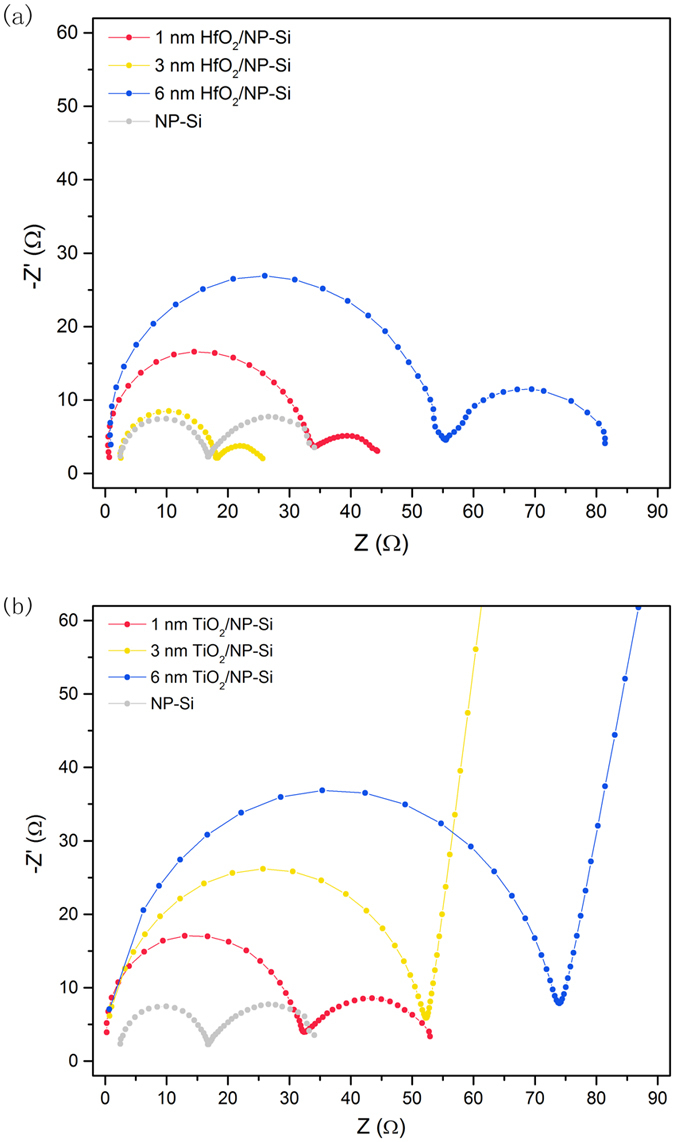
Electrochemical impedance spectroscopy of the NP-Si (gray), the HfO_2_/NP-Si (**a**) and the TiO_2_/NP-Si (**b**) with the different thickness x under illumination. Frequency is from 1 × 10^6^ to 0.1 Hz. x = 1 (red), 3 (yellow), 6 (blue) nm.

Figure 5 is the EIS of the NP-Si, the HfO_2_/NP-Si, and the TiO_2_/NP-Si with the different thicknesses of passivation layers. The EIS reflects the obstruction of charge carriers’ transportation from electrode to reactants in solution. In EIS, more attentions are paid to the front semicircle rather than the following arc or the oblique line, because the former belongs to electrochemical process at high frequency zone, and the latter corresponds to reactants diffusion or absorption processes at low frequency zone. As shown in Fig. 5(a), for the HfO_2_/NP-Si, with increasing the thickness of HfO_2_, the radius of the front semicircle, i.e. the resistance of electrochemical reaction R_r_, decrease firstly and increase subsequently. The R_r_ reflects carriers transport from the deep in Si to the interface of electrode/electrolyte for reacting with reactants. And the recombination at the interface of HfO_2_/NP-Si and tunneling effect will affect R_r_. With increasing the thickness of HfO_2_ layer, the recombination rate of carriers is decreased, however the tunneling effect is also weakened. Hence, there is a trade-off between the two effects. The smallest R_r_ of the HfO_2_/NP-Si is obtained when the thickness of HfO_2_ layer is 3 nm, however it is still a slight larger than that of the NP-Si. While for the TiO_2_/NP-Si, the R_r_ keep decreasing with increasing the thickness of TiO_2_. The changes of R_r_ are consistent with the variation of photocurrent densities as shown in Fig. 3(a) and (b), respectively. It is worth noting that the arc change into an oblique line after the thickness of TiO_2_ is larger than 1 nm. This is attributed to the change of reactants diffusion zone in solution. When the speed of carriers transport for reacting with reactants at the interface of electrolyte/electrode (V_ct_) is faster than the speed of reactants diffusion from the region far away from interface to the interface (V_rd_), the V_rd_ is equal to the limitation of the diffusion speed of reactants. Then the reactants diffusion zone will keep a fixed thickness to support an ultimate V_rd_. In the meantime, the front semicircle curve extends to an arc in EIS. When the V_ct_ is slower than the V_rd_, the reactants diffusion zone in solution turns into a layer that varies with the V_ct_, then the arcs turn into the oblique lines in EIS.

**Figure 6:**
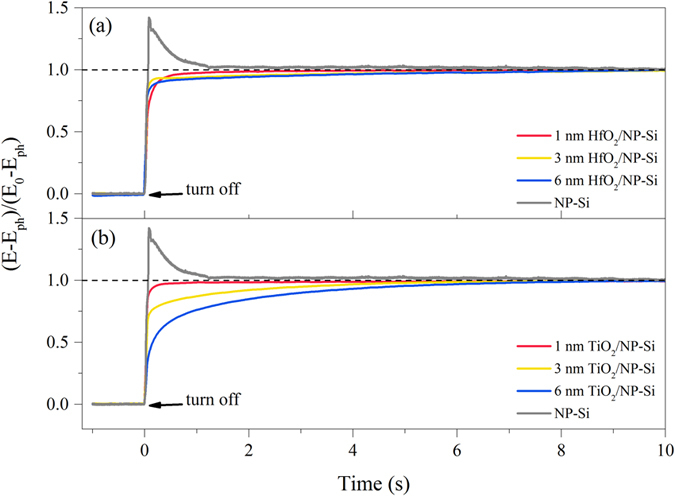
Normalized open-circuit potential (OCP) decay curves of the NP-Si (gray), the HfO_2_/NP-Si (**a**) and the TiO_2_/NP-Si (**b**) with the thickness of 1 (red), 3 (yellow), 6 (blue) nm, respectively.

The amorphous HfO_2_ or TiO_2_ layer deposited by ALD could facilitate the extraction of charge carriers, and increase the water splitting efficiency by reducing interface defects. This is because the defects at interface are the main traps to capture charge carriers. The captured charge carriers are easy to recombine with the photogenerated carriers. The coating of passivation layers on NP-Si can effectively reduce the number of defects and suppress the recombination of charge carriers. The accumulation of photogenerated carriers at the interface can be measured as a function of the open-circuit potential (OCP). Thus, the density of defects at interface will be reflected from the decay speed of OCP. The normalized transient decay profiles of open-circuit potential (OCP) are displayed in Fig. 6, which can measure the passivation effect at the interface between the passivation layers and NP-Si. An average recombination rate constant *k*_*τ*_ is obtained by fitting the decay profiles with a first-order kinetic mode. The mode equation is as following^19^:


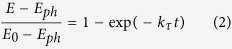


Where 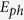 is the stationary OCP under illumination, 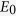 is the stationary OCP without illumination. However, on account of the enormous amount of defects on the surface of the NP-Si, the photogenerated carriers rapidly combine with the captured charge carriers in a remarkably short period of time (<0.08 s) when the light is turned off. Then the defects rapidly capture a large amount of thermal excitations carriers, which elevates the open-circuit potential to a stable level in a relative short period of time (~1.2 s). As a result, a sharp protuberance curve is presented in the decay profile of OCP as shown in Fig. 6. The passivation layers are the conformal layers, which can effectively reduce the number of defects. Even so, the recombination at surface still fast in the NP-Si, the changes of OCP are very weak after 10 s, due to complex geography that caused by nanoporous structure. With the increase of layer thickness, the decay curves become more and more relaxative in both the OCP decay profiles of the HfO_2_/NP-Si and the TiO_2_/NP-Si. For the decay speed, a more obvious relaxation is presented in the OCP decay profile of the TiO_2_/NP-Si (Fig. 6(b)), the recombination rate of 6 nm TiO_2_/NP-Si (*k*_*τ*_ = 1.63) is around 15 times smaller than that of 6 nm HfO_2_/NP-Si (*k*_*τ*_ = 23.96). The passivation ability has a small change with increase in the thickness of the HfO_2_ layer, while it is promoted with increase in the thickness of TiO_2_ layer.

### Stability

**Figure 7:**
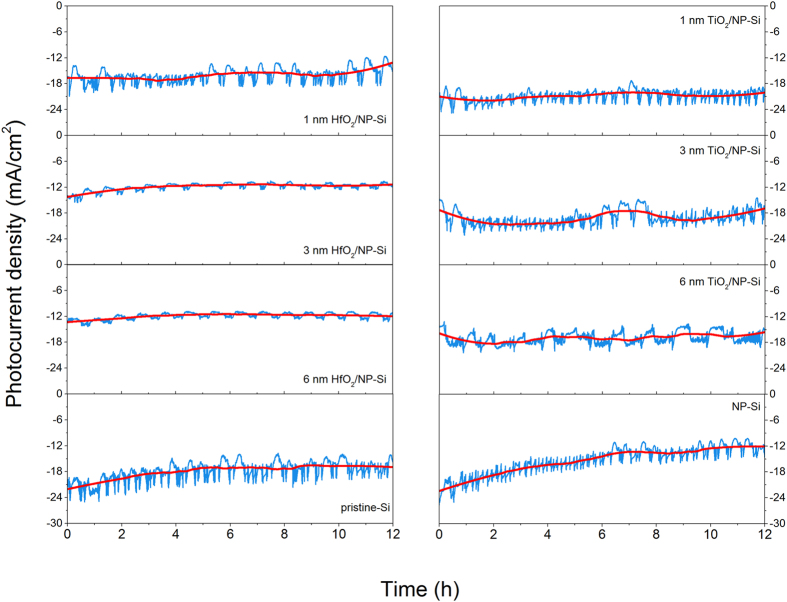
I-t curves (12 h) of the HfO_2_(x)/NP-Si, the TiO_2_(x)/NP-Si, the pristine-Si, and the NP-Si at −0.8 V vs. Ag/AgCl. The thickness of passivation layers x = 1, 3, 6 nm. The red lines are the corresponding smoothed curves.

Figure 7 is the stability measurements between the pristine-Si, the NP-Si, the HfO_2_/NP-Si and the TiO_2_/NP-Si with the different thicknesses of passivation layers. There are fluctuations for the photocurrent densities. We ascribe the fluctuations to the desorption of the gas and intensity oscillation of incident light. Then, the smoothed lines (the red line in Fig. 7) are used to analysis the changes of the photocurrent densities. The photocurrent density of NP-Si is greatly decrease from 22.4 to 12.1 mA/cm^2^ after 12 hours illumination, and the decay ratio of photocurrent density is 46%, which is higher than the 15.5% of the pristine-Si. Although the photocurrent density of the NP-Si increases comparing to that of the pristine Si, the stability becomes worse. Because of its large surface to volume ratio, the photocurrent density of the NP-Si will decrease sharply, when the layer thickness of SiO_x_ exceeds the effective thickness of tunneling effect after long time light illumination. Covering HfO_2_ layers with the thicknesses from 1 to 6 nm, the photocurrent density decay ratio decreases from 21.3% to 10.3%. While, the decay of the photocurrent densities of the TiO_2_/NP-Si are very small. The 4.4% of photocurrent density decay is presented in the 1 nm TiO_2_/NP-Si, an only 1.9% is presented in the 6 nm TiO_2_/NP-Si. In order to further analysis the stability of different kind of passivation layers, another 48 h I-t curves between the 6 nm HfO_2_/NP-Si, the 6 nm TiO_2_/NP-Si and the NP-Si were measured, as shown in supplementary material. During the 48 h measurement, the photocurrent density of the NP-Si keeps deceasing from 13.7 to 11.0 mA/cm^2^. The photocurrent density of the 6 nm HfO_2_/NP-Si decreases in the first 4 h, and keeps stable in the last 44 h. While, the photocurrent density of the 6 nm TiO_2_/NP-Si stabilized at 12 mA/cm^2^ during the whole 48 h. These measurements show that the stability of TiO_2_ as passivation layer in aqueous is better than that of HfO_2_ with the same thickness.

## Discussion

For the HfO_2_/NP-Si, the saturated photocurrent density is nearly 6 mA/cm^2^ higher than that of NP-Si when the thickness of HfO_2_ layer is 3 nm. It is believed that the higher photocurrent densities benefit from the field effect passivation and the chemical passivation of the HfO_2_ layer^20^. The field effect passivation come from the positive fixed charge in HfO_2_. Sreenivasan *et al*. reported that the density of positive fixed charge in HfO_2_ is as high as 4.5 × 10^11^/cm^2^ 21. The chemical passivation is the effect that reduces the defects at the surface of NP-Si. The change of saturated photocurrent densities that increase firstly then decrease with increase in the thickness of HfO_2_ layer is a balance between the increase of the passivation effects and decrease of the tunneling effect, which agrees with the result reported by M. Jin Choi *et al*.^14^.

While, with increase of the thickness of the HfO_2_ layer, the change of the R_r_ in Fig. 5(a) is corresponding to the change of the saturated photocurrent densities in Fig. 3(a), which is the reflection of the comprehensive passivation including field effect passivation and chemical effect passivation. However, the change of open-circuit potential decay curves of the HfO_2_/NP-Si (Fig. 6(a)), which reflects the chemical effect passivation of the defects at the interface, is not obvious, and it does not identify with the change of corresponding saturated photocurrent densities (Fig. 3(a)). Therefore, the field effect passivation is believed to be the dominating factor for the passivation effect in the HfO_2_/NP-Si.

As shown in Fig. 3(b), saturated photocurrent densities decrease obviously with increasing thickness of the TiO_2_ layer, and are lower than that of the NP-Si. A depletion layer will be formed at the interface of electrolyte/TiO_2_, which impedes the transfer of electrons from TiO_2_ to electrolyte. However, at the other interface of the TiO_2_/NP-Si, a depletion layer that facilitates electrons transfer to TiO_2_ will be formed, due to the p-n heterojunction. If the TiO_2_ is thin enough, the two kinds of depletion layer will overlap to form a space charge layer, and electrons can diffuse to the electrolyte directly. However, with increasing thickness of TiO_2_ layer, the thickness of the overlapped space charge layer will decrease until to form an electron trap, which is similar to a back-to-back diode. This electron trap is the main obstacle of electrons transfer, whereas, the photogenerated carriers can reduce the obstacle when the TiO_2_ is under illumination.

The electrical resistivity of p-Si in this work is about 5 Ω∙cm, the corresponding doping density is about 1.53 × 10^15^ cm^−3^, which is considered to be the concentration of majority carriers (holes). However, the measured carrier concentration in the NP-Si is 6.7 × 10^14^ cm^−3^ (Fig. 4). This deviation is attributed to the high density of surface states. After deposited a passivation layer, the carrier concentration of the HfO_2_/NP-Si and the TiO_2_/NP-Si are back to the range of 10^15^ cm^−3^.

**Figure 8:**
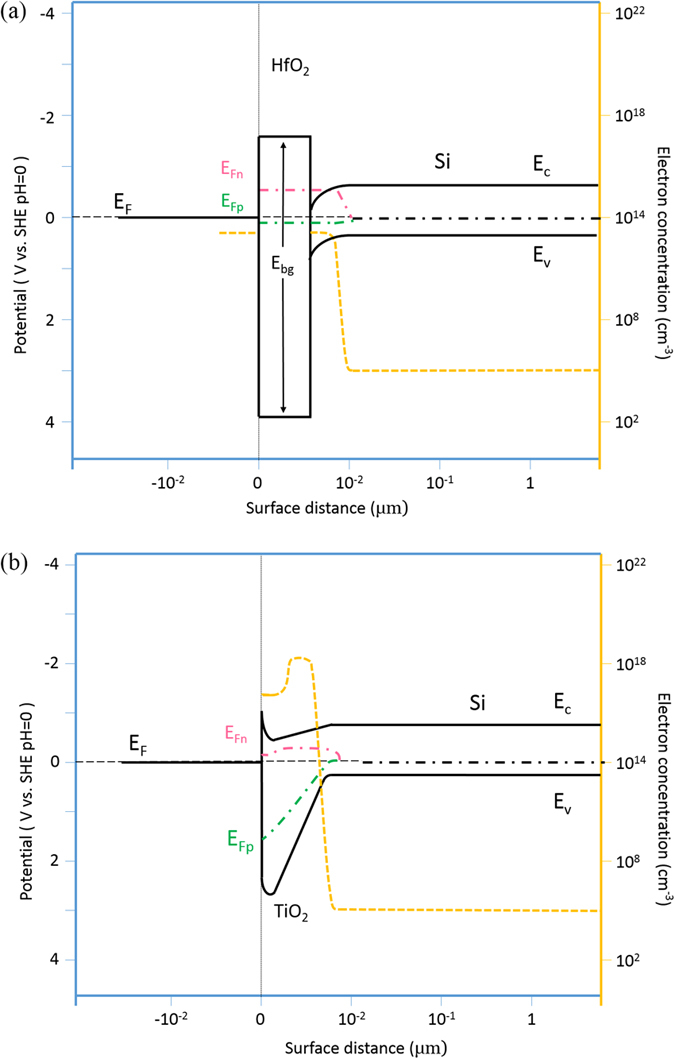
The band schemes and electron concentrations of HfO_2_/NP-Si (**a**) and TiO_2_/NP-Si (**b**). E_Fn_ and E_Fp_ are electron quasi-Fermi level and hole quasi-Fermi level under illumination.

According to the above discussion, the band schemes and the corresponding electron concentrations under illumination of the HfO_2_/NP-Si and the TiO_2_/NP-Si are proposed in Fig. 8(a) and (b), respectively. The energy gap of HfO_2_ is 5.7 eV, and the CB offset and VB offset are 1.54 eV and 3.04 eV, respectively^22^. The CB and VB bending are due to the build-in electrical field at the interface between HfO_2_ and NP-Si produced by the fixed charges in HfO_2_. The different band offsets in the HfO_2_/NP-Si will facilitate the transfer of electrons, and block the transfer of holes. The concentration of minority carriers (electrons) in the deep of the NP-Si is ~10^5^ cm^−3^. When the illumination is supplied, it will be elevated to 2~5 × 10^13^ cm^−3^ within the absorption depth of light^23^, while the concentration of majority carriers (holes) has a small change. Then, the Femi level within the absorption depth of light splits into a higher electron quasi-Fermi level that close to the CB of the NP-Si, and a hole quasi-Fermi level that close to the original Femi level of the NP-Si.

As shown in Fig. 8(b), electron concentration in the NP-Si is promoted by TiO_2_ that possesses high intrinsic electrons (~10^18^ cm^−3^) due to the existence of oxygen vacancies. Then electron concentration decreases a little at the distance that further close to the surface, this is attributed to the depletion layer at interface of the electrolyte/TiO_2_. The Femi level of the TiO_2_/NP-Si starts to split since deep in the depletion layer at the interface of the TiO_2_/NP-Si. The hole quasi-Fermi level is close to the VB of TiO_2_, and be promoted under illumination, while the transfer of holes from NP-Si to TiO_2_ is blocked because of the large VB offset of the TiO_2_/NP-Si. The electron quasi-Fermi level in TiO_2_ is higher than the original Femi level of Si, and bends down to a more negative level due to the depletion layer at interface between electrolyte and TiO_2_. The two depletion layers at the interfaces of electrolyte/TiO_2_ and TiO_2_/NP-Si overlap to form a successive space charge layer (Fig. 8(b)).

Although the saturated photocurrent density of the NP-Si is relatively large due to the nanoporous structure, its stability is not satisfactory. However, the increase of the stability for the NP-Si covered both HfO_2_ and TiO_2_ passivation layers is obvious. Comparing the HfO_2_/NP-Si and the TiO_2_/NP-Si, the later has higher stability. The photocurrent density of the 6 nm TiO_2_/NP-Si only decreases 1.9% during the first 12 h measurement, and shows an excellent stability during the following 48 h measurement.

In conclusion, we compared the passivation effects, the PEC properties, and studied the corresponding mechanisms. The saturated photocurrent densities of HfO_2_/NP-Si are higher than that of TiO_2_/NP-Si. This phenomenon is due to the advantages of the field effect passivation and the chemical passivation from the HfO_2_ layers and the disadvantage of the electron trap that formed by the overlapping of two space charge layers at the electrolyte/TiO_2_ and the TiO_2_/NP-Si. However, the long-term I-t measurements demonstrate that the stability of TiO_2_/NP-Si is better than that of HfO_2_/NP-Si with the same thickness of passivation layer.

## Methods

Before metal-assisted chemical etching (MaCE), B-doped p-type <100> single face polished Si wafer with electrical resistivity of 1–10 Ω·cm was cleaned by RCA (Radio Corporation of America) method, and dried under N_2_. Later on, 8 nm thick Au layer deposited on the pre-cleaned Si wafer, followed by annealing at 600 °C in Ar for 1 h. Au nanoparticle arrays were formed on the surface of Si wafer. Then, the Au nanoparticles coated sample was dipped into 2.73 M HF and 1.32 M H_2_O_2_ MaCE solution for 10 min, followed by cleaning with DI water and drying under N_2_. Nanoporous structure was formed due to Au nanoparticles catalyzed etching of Si. 0.5% wt. tetramethylammonium hydroxide (TMAH) was used to polish the surface of NP-Si for 5 s.

To avoid the formation of SiO_x_ layer between Si and passivation layer, the samples were dipped into 5% wt. HF solution before ALD, then dried and transferred to the vacuum chamber as soon as possible. HfO_2_ was grown using tetrakis (dimethylamido) hafnium as Hf source and H_2_O as the oxidizer at 95 °C. The growth rate was about 0.122 nm/cycle and the films were grown for 8, 25, and 50 cycles. TiO_2_ was grown using tetrakis (dimethylamido) titanium as Ti source and H_2_O as the oxidizer at 125 °C. The growth rate was about 0.05 nm/cycle and the films were grown for 20, 60, and 120 cycles. The corresponding thicknesses of the TiO_2_ and the HfO_2_ layers are 1, 3, 6 nm, respectively.

Scanning electron microscopy (SEM) was used to characterize the surface morphology and the thicknesses of samples. Reflection spectra were measured by UV-vis-near-IR spectrophotometer (Hitachi U-4100). Three electrodes cell was used to measure the PEC properties under 100 mW/cm^2^ illumination using 500 W Xe lamp with an AM 1.5 optical filter as the light source. The polytef cell outfitted an Ag/AgCl (saturated KCl) electrode as the reference electrode and a 20 cm platinum wire as the counter electrode. The electrolyte of 0.5 M H_2_SO_4_ was circulated by a peristaltic pump. A piece of copper tape was adhered by Ag paste and In-Ga alloy to form back ohmic contact.

## Additional Information

**How to cite this article:** Xing, Z. *et al*. Enhanced PEC performance of nanoporous Si photoelectrodes by covering HfO_2_ and TiO_2_ passivation layers. *Sci. Rep.* **7**, 43901; doi: 10.1038/srep43901 (2017).

**Publisher's note:** Springer Nature remains neutral with regard to jurisdictional claims in published maps and institutional affiliations.

## Supplementary Material

Supplementary Material
