# Supplementary material for: Enhanced PEC performance of nanoporous Si photoelectrodes by covering HfO2 and TiO2 passivation layers
Source: Sci Rep. 2017 Mar 2;7:43901. doi: 10.1038/srep43901 (PMC5333152; doi:10.1038/srep43901)

# Enhanced PEC performance of nanoporous Si photoelectrodes by covering HfO_2_ and TiO_2_ passivation layers

Zhuo Xing^1^, Feng Ren^*1^, Hengyi Wu^1^, Liang Wu^1^, Xuening Wang^1^, Jingli Wang^2^, Da Wan^2^, Guozhen Zhang^2^, Changzhong Jiang^1^

1. Center for Ion Beam Application and Center for Electron Microscopy, School of Physics and Technology, Wuhan University, Wuhan 430072, People’s Republic of China.
2. Key Laboratory of Artificial Micro- and Nano-structures of Ministry of Education, School of Physics and Technology, Wuhan University, Wuhan 430072, People’s Republic of China.

* Corresponding author. Tel: +86-27-68752567 Fax: +86-27-68752569

Email address: fren@whu.edu.cn

**Supplementary information:** I-t curves of the 6 nm HfO_2_/NP-Si, the 6 nm TiO_2_/NP-Si, and the NP-Si for supplementary 48 hours measurements at -0.8V vs. Ag/AgCl.
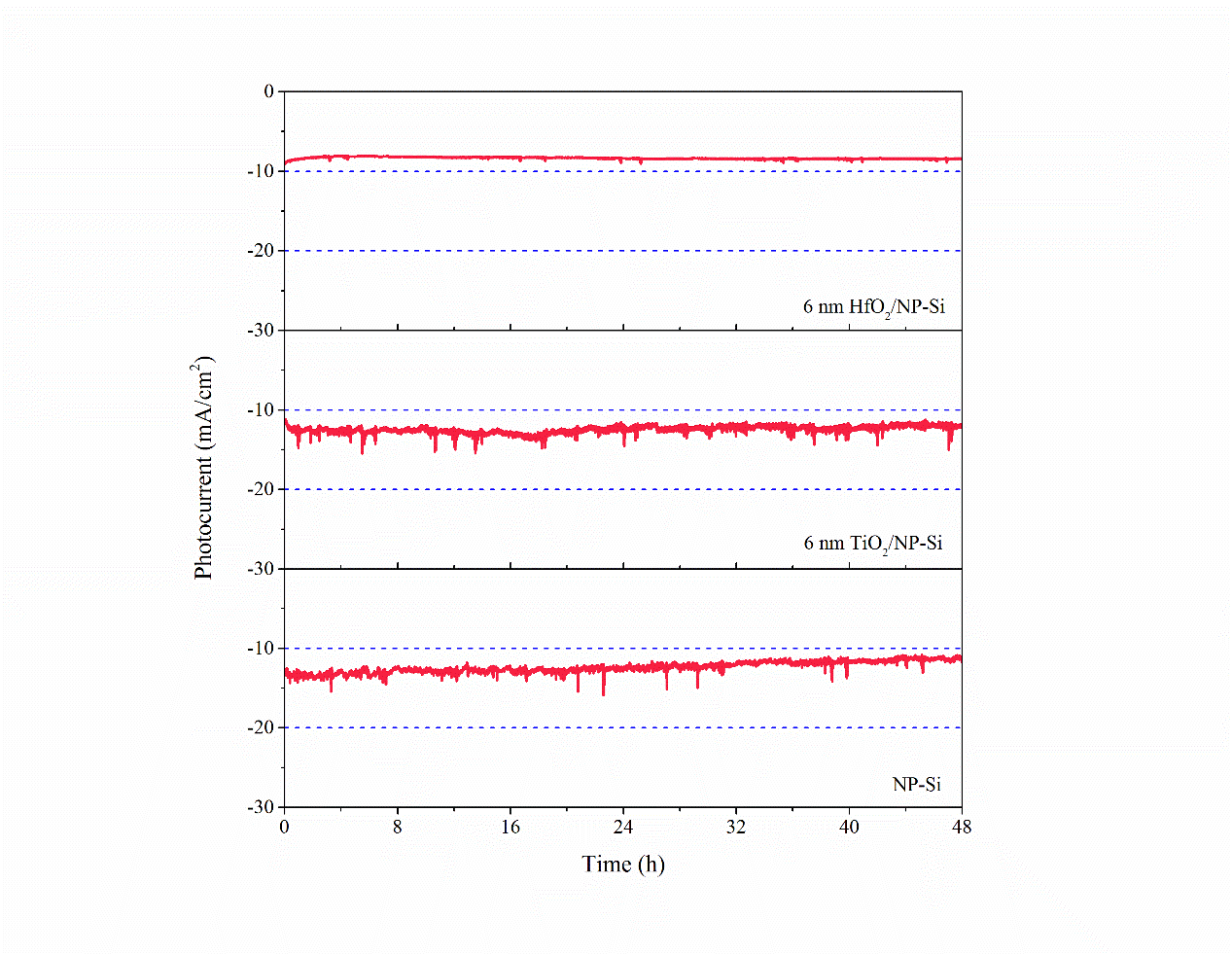

Supplement: Supplementary Material [file srep43901-s1.doc]
